# A Design Method for an SVM-Based Humidity Sensor for Grain Storage

**DOI:** 10.3390/s24092854

**Published:** 2024-04-30

**Authors:** Lining Liu, Chengbao Song, Ke Zhu, Pingzeng Liu

**Affiliations:** 1 College of Mechanical and Electronic Engineering, Shandong Agricultural University, Tai’an 271018, China; nk18754882706@163.com (L.L.); ziwei_s1@163.com (C.S.); 2 Key Laboratory of Huang-Huai-Hai Smart Agricultural Technology, Ministry of Agriculture and Rural Affairs, Tai’an 271018, China; 3 College of Information Science and Engineering, Shandong Agricultural University, Tai’an 271018, China

**Keywords:** humidity sensor, grain moisture, standing wave ratio (SWR) method, moisture calibration models

## Abstract

One of the crucial factors in grain storage is appropriate moisture content, which plays a significant role in reducing storage losses and ensuring quality. However, currently available humidity sensors on the market fail to meet the demands of modern large-scale grain storage in China in terms of price, size, and ease of implementation. Therefore, this study aims to develop an economical, efficient, and easily deployable grain humidity sensor suitable for large-scale grain storage environments. Simultaneously, it constructs humidity calibration models applicable to three major grain crops: millet, rice, and wheat. Starting with the probe structure, this study analyzes the ideal probe structure for grain humidity sensors. Experimental validations are conducted using millet, rice, and wheat as experimental subjects to verify the accuracy of the sensor and humidity calibration models. The experimental results indicate that the optimal length of the probe under ideal conditions is 0.67 m. Humidity calibration models for millet, rice, and wheat are constructed using SVM models, with all three models achieving a correlation coefficient R^2^ greater than 0.9. The measured data and model-calculated data show a linear relationship, closely approximating y = x, with R^2^ values of all three fitted models above 0.9. In conclusion, this study provides reliable sensor technological support for humidity monitoring in large-scale grain storage and processing, with extensive applications in grain storage and grain safety management.

## 1. Introduction

The advancement of modern information technology, particularly sensor technology, has presented significant opportunities for the progression of traditional agriculture. The adoption and widespread use of modern agricultural equipment [1,2,3,4,5,6,7,8,9,10] have not only transformed the production management and operational methods of traditional agriculture but have also increased the efficiency and yield of high-quality agricultural products. Grain storage, as an essential component of food security, holds immense importance for strategic commodity reserves and ensuring food security. However, statistical data from 2020 indicate that the private grain storage loss rate in China reached as high as 8%. Therefore, achieving modernized management of grain storage, particularly by enhancing the accuracy of perception and management of the internal storage environment, is crucial for extending the storage lifespan of grains and mitigating issues such as mold growth, fermentation, and shrinkage.

The humidity level in grain storage plays a crucial role in maintaining the quality of stored grain, which impacts factors such as grain nutrition, grading quality, and the prevention of mold growth and fermentation [11,12,13,14]. Excessive humidity will create a damp environment conducive to mold growth, leading to grain spoilage. However, excessively low humidity will cause grains to crack and dry out. Both scenarios adversely affect grain reserves and impeding national food security efforts. Therefore, humidity levels in grain storage environments, particularly in large-scale facilities, must be maintained within an appropriate range [15]. This is essential for prolonging effective storage duration and preserving grain quality. Although safe storage humidity may vary for different grains, it typically falls within the range of 10% to 14% [12,16]. Addressing these challenges requires the development of an economical, highly accurate, and easily scalable grain humidity monitoring sensor device suitable for large-scale grain storage. This device would facilitate real-time acquisition of humidity information in various grain storage environments. The paper proposes the development of a grain humidity sensor capable of real-time perception of grain moisture content. It needs to provide fundamental data for environmental control systems within storage facilities. Additionally, employees can analyze the quality status of stored grain based on historical humidity information.

The monitoring of grain humidity demands sensors with high standards in sensing accuracy, effective monitoring range, durability, stability, anti-interference capability, and cost-effectiveness. Currently, humidity monitoring technologies commonly fall into two categories: contact and non-contact. Non-contact technologies, such as those utilizing microwave [17,18,19,20], infrared [21,22], or ultrasonic waves, enable moisture measurement without direct contact with the medium. While these methods avoid damaging the medium, their sensing accuracy typically lags behind that of contact methods, and they often involve higher costs. Contact humidity sensing devices [23,24,25,26,27] are better suited to fulfilling the requirements of the domestic large-scale grain storage industry concerning humidity monitoring accuracy, equipment maintenance, and system scalability.

Researchers have developed various types of contact humidity sensors. They can accurately collect humidity information from different mediums such as soil [21,22,23], grains [24,25,26,27], plants [28], and chemical materials [29]. Contact humidity sensors are categorized into different types based on their principles, including capacitance principle, resistance principle, time-domain reflectometry (TDR), frequency domain reflectometry (FDR), and standing wave ratio (SWR) [30]. 

Capacitive humidity sensors [31,32] on the market are predominantly of the insertion type. They acquire analog information about moisture by embedding probes into the medium. Then, specific humidity calibration models are employed to convert the perceived analog information into actual moisture data. For example, a capacitive humidity sensor applies square wave excitation signals to a first-order RC circuit composed of resistors, probes, and the medium under test. Then it measures the periodic fluctuations on the probe to obtain humidity information about the medium [30].

A resistive humidity sensor treats the medium under test as a humidity-dependent variable resistor. Humidity information about the medium is obtained by measuring the voltage across the probe terminals [30].

The theoretical foundation of the TDR moisture sensor draws from research conducted by Fellner-Feldegg [33] et al. The signal generator of the TDR humidity sensor emits a pulse signal. As this signal travels through the coaxial cable to the probe, an impedance mismatch occurs. A portion of the signal reflects back along the original path, while the rest continues to propagate along with the probe. The detection device measures the time difference between these two reflected signals, which corresponds to the time it takes for the signal to travel twice along with the probe [30].

The FDR humidity sensor utilizes the medium as a dielectric, with the sensor’s probe acting as a capacitor alongside an external oscillator. This setup forms a tuning circuit during the measurement process. The capacitance of the sensor is directly related to the dielectric constant of the medium being measured between the two levels. As moisture levels rise, the equivalent capacitance of the sensor also increases, thereby affecting the sensor’s operating frequency (resonant frequency) [30,34].

An SWR humidity sensor’s signal generator produces pulses. When the signal is transmitted to the probe, some parts continue along with the probe while others return along the original path. At this point, the incident and reflected signals superimpose, forming standing waves. It retrieves humidity information by measuring the standing wave ratio between two reflected signals [35]. The voltage difference of this sensor varies with changes in the probe’s impedance, which is determined by the medium’s dielectric constant [36].

**Figure 1:**
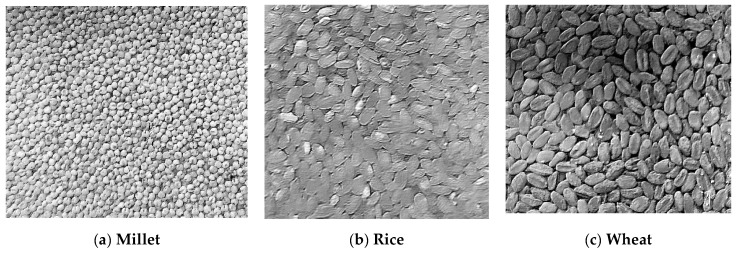
The longitudinal profile of grain storage.

**Figure 2:**
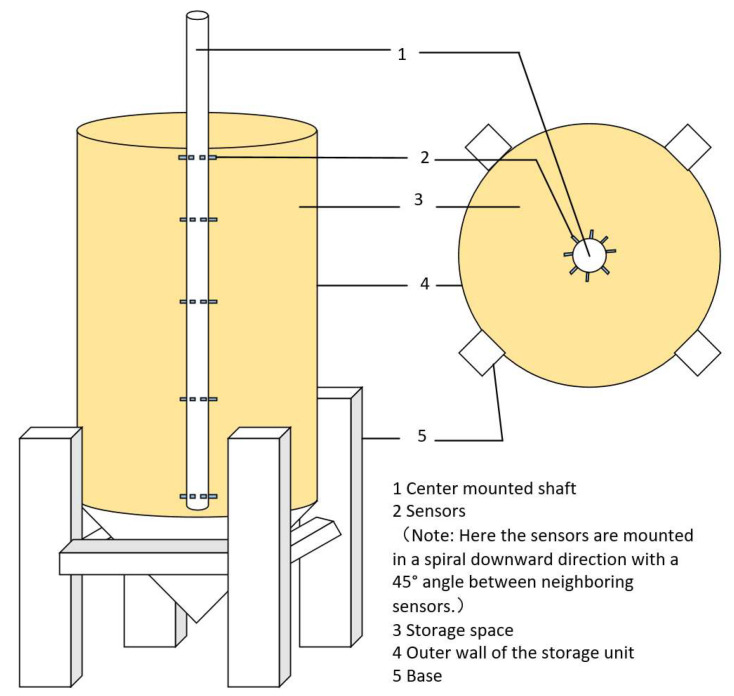
The installation diagram of the sensor.

In real life, the humidity sensors are widely applied in various mediums, such as rice, maize, corn, black beans, etc. However, the calibration models provided by manufacturers are typically fixed to one or a few types. Due to variations in the volume and shape of different grain mediums, their moisture absorption capacities differ. (The cross-sectional structure of grains in storage is shown in Figure 1. We placed the grains into a transparent square container and captured images from the side of the container. Through image processing techniques, binary images were obtained to highlight the gaps between the grains in the medium, allowing Figure 1 to be ultimately obtained. And the installation diagram of the sensor is shown in Figure 2.) Relying on a single humidity calibration model may lead to inaccuracies in capturing humidity data across different grains. Moreover, there are subtle variations in the ideal storage humidity levels for various grains. For instance, millet, wheat, rice, and corn exhibit optimal storage humidity levels of approximately 12%, 12–14%, around 14%, and 13–14%, respectively. Consequently, using a singular humidity calibration model cannot effectively cater to the precise monitoring of storage humidity for multiple grains, even impeding the meticulous management of diverse grain storage environments. To tackle this challenge, this study conducts an analysis of the distinct characteristics of various grains and proposes tailored humidity calibration models specifically designed for three common major grains: millet, rice, and wheat. These customized models significantly enhance the versatility and precision of humidity sensors. Leveraging the SWR principle, the developed sensors dynamically adjust the humidity calibration model based on the grain type, thereby ensuring the accuracy of the final computation results. Furthermore, upon detecting that the humidity of the grain medium has reached the storage humidity warning threshold, the system initiates an alert, prompting users to adjust the ventilation equipment in the storage facility accordingly. Additionally, the system can collaborate with ventilation equipment controllers to achieve automated and intelligent management of storage facilities.

In this study, we have developed a grain moisture monitoring humidity sensor utilizing the SWR principle to fulfill the need for precise humidity monitoring across various grain types. We conducted both theoretical analysis and experimental validation of humidity calibration models specific to different grain varieties. Subsequently, we put forward three tailored humidity calibration models for millet, wheat, and rice, respectively. These models successfully enabled accurate monitoring of storage humidity levels for these key grain types. This approach effectively resolves the practical challenge of inadequate grain storage practices stemming from the limitations of applying a single humidity calibration model to detect humidity across diverse grain types. The structure of this paper is as follows: Part II provides an overview of current research on relevant moisture sensors. Part III outlines the principles underlying the sensors and associated models. Part IV presents the design and simulation experiments of the sensors. Part V details the validation experiments and analysis of results. Part VI summarizes the advantages and limitations of the developed sensing and moisture calibration model, while also suggesting avenues for future improvements.

## 2. Related Work

Humidity monitoring technology can be categorized into two types based on the operation: contact and non-contact. Non-contact techniques primarily utilize microwave [17,18], infrared [19,20], or ultrasonic technologies, enabling moisture measurement without directly contacting the medium. Although non-contact methods do not damage the medium, their sensing accuracy is lower compared to contact methods, and they generally incur higher costs. Contact humidity sensing devices [21,22,23,24,25,26,27] offer lower costs and higher sensing accuracy. Therefore, they are better suited to meet the demand for grain humidity monitoring in the domestic large-scale grain storage industry.

The non-contact humidity sensor acquires moisture information from the medium by receiving and transmitting antenna-specific wavelength information. It is typically used for humidity monitoring of small-scale, high-precision products. For instance, Jiarasuwan S. et al. [37] developed a microwave moisture sensor suitable for small irregular containers for grains. Experiments were conducted with various grains such as rice, red beans, black beans, and containers of different shapes, verifying the accuracy of moisture monitoring by the sensor. Additionally, Singh D.K. et al. [38] proposed a microstrip-coupled line humidity sensor. It utilizes a vector network analyzer to measure reflection coefficients, thereby improving the detection accuracy of broken rice humidity.

Compared to non-contact humidity sensors, contact humidity sensors offer the advantages of high precision and low cost, making them more suitable for large-scale grain storage applications. Common detection principles include resistive, capacitive, thermoelectric, and surface acoustic wave principles. Resistive sensors have lower sensitivity and larger dimensions. Thermoelectric and surface acoustic wave sensors offer high accuracy but are more costly and susceptible to interference from other factors, making them unsuitable for use in large grain storage facilities. Additionally, vendors typically provide only one humidity calibration model [39,40,41,42,43], which requires users to independently conduct experiments to adjust the humidity calibration model when encountering different mediums. This imposes higher demands on cost and time. For example, Thakur R. et al. [25] developed a capacitive sensor and a corresponding moisture calibration model for multiple grains by optimizing sensor circuits. This enables efficient monitoring of mustard, chickpea, wheat, and other grain moisture levels. Lei X. et al. [21] conducted calibration experiments on capacitive humidity sensors with different bulk densities of soil and optimized the calibration model, improving the correlation between sensor readings and soil moisture content. This allows the sensor to more directly reflect specific values of water content and humidity trends in soil columns. Bogena H.R. et al. [43] proposed a dedicated calibration method for the smt100 sensor, which improves calibration accuracy while simplifying calibration time.

This paper aims to design a cost-effective contact-type grain moisture sensor while constructing humidity calibration models based on the major domestically stored grains such as millet, rice and wheat. To achieve this goal, the paper provides a detailed explanation of the design principles and experimental processes of both the sensor and the models. Additionally, validation experiments for the developed sensor’s humidity calibration models are designed, and the advantages of developing the sensor are analyzed.

Table 1 presents the principles and models of some humidity sensors.

## 3. Sensor Principles and Model Analysis

### 3.1. Moisture Measurement Principle Based on SWR

Grains in storage can be considered as a mixture of grain, air, and water. The dielectric constants of grain and air are relatively low (approximately 4 for grain and about 1 for air), while water has a dielectric constant of approximately 80. Therefore, the comprehensive dielectric constant of grains varies with the moisture content of the grains. Thus, we can utilize the dielectric constant of grains to infer their moisture content. Detection methods of this kind can be categorized into various types based on principles, such as capacitive method, resistive method, and SWR method, among others [36]. After comprehensive consideration of the technical difficulty, accuracy, and economic cost of sensor information acquisition, we ultimately chose SWR as the principle.

**Figure 3:**
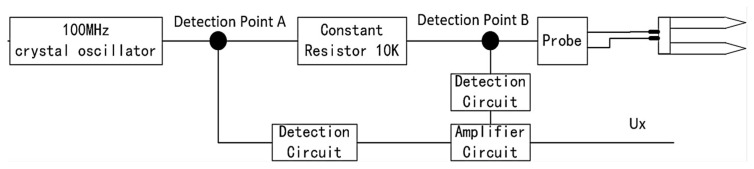
The structural design of the circuit.

Research has shown that the accuracy of measuring the dielectric constant of grains is sensitive to the measurement frequency. When the measurement frequency is below 100 MHz, the presence of salts in the grain medium can significantly interfere with the measurement results of the dielectric constant. However, when the measurement frequency ranges between 100 to 500 MHz, factors such as salts have minimal interference with the measurement results of the dielectric constant [49,50]. Based on a comprehensive consideration of sensor power consumption and data processing capabilities, this study ultimately adopted 100 MHz as the measurement frequency for the sensor. The sensor structure is illustrated in Figure 3. As depicted in the figure, the measurement circuit primarily consists of a crystal oscillator, fixed resistors, and a probe. The dielectric constant $\varepsilon_{m}$ of the sensor probe is a fixed value. The dielectric constant $\varepsilon_{i}$ of the grains varies with the moisture content. Therefore, the relative equivalent dielectric constant $\varepsilon_{x}$ of the probe is jointly determined by $\varepsilon_{m}$ and $\varepsilon_{i}$. The theoretical design calculation formula for the equivalent medium is as follows:

(1) $$\varepsilon_{x}=\varepsilon_{m}*(1+\varphi *(\varepsilon_{i}-\varepsilon_{m})/(\varepsilon_{m}+2*\varphi (\varepsilon_{i}-\varepsilon_{m})))$$

In the equation, *φ* represents the filling factor, indicating the proportion of material occupied in the probe. It can be obtained through experimental or simulated calculations. Additionally, there exists the following relationship between the equivalent dielectric constant and impedance:

(2) R=1/(jωC)

R represents impedance, *ω* is the angular frequency, *j* is the imaginary unit, and *C* represents the capacitance of the grain. The capacitance value of the grain can be expressed by the dielectric constant *ε* and geometric data, such as electrode spacing and electrode area.

(3) $$C=\varepsilon_{x}*A/d$$

Here, *A* represents the area of the probe, and *d* represents the separation of the probe. If the sum of the fixed-value resistor and the circuit impedance in the circuit is denoted as $R_{0}$(Ω) and the equivalent input impedance of the probe after inserting the grain is denoted as $R_{x}$(Ω). The relationship between voltage and impedance can be expressed as:

(4) $$U_{x}=K_{f}(U_{A}-U_{B})$$

(5) $$R_{x}=(R_{0}*U_{B})/(U_{A}-U_{B})$$

(6) $$U_{x}=(K_{f}*U_{B}*R_{0})/R_{x}$$

In Formula (4), $K_{f}$ represents the amplification factor of the amplification circuit. In Formula (6), it can be observed that when the moisture content of the granular medium changes. In addition, the output voltage $U_{x}$ of the sensor will correspondingly change. Therefore, the numerical value of the output voltage can be utilized to deduce information about the humidity of the grain medium.

### 3.2. Humidity Calibration Model

**Figure 4:**
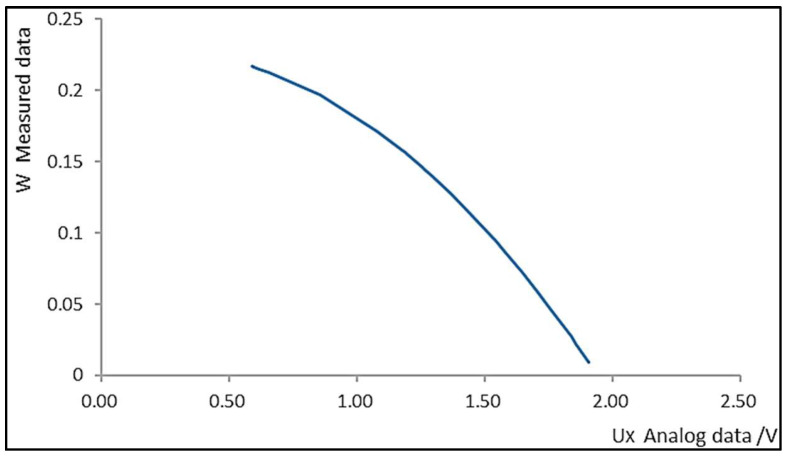
The voltage of sensor at different humidity levels.

**Figure 5:**
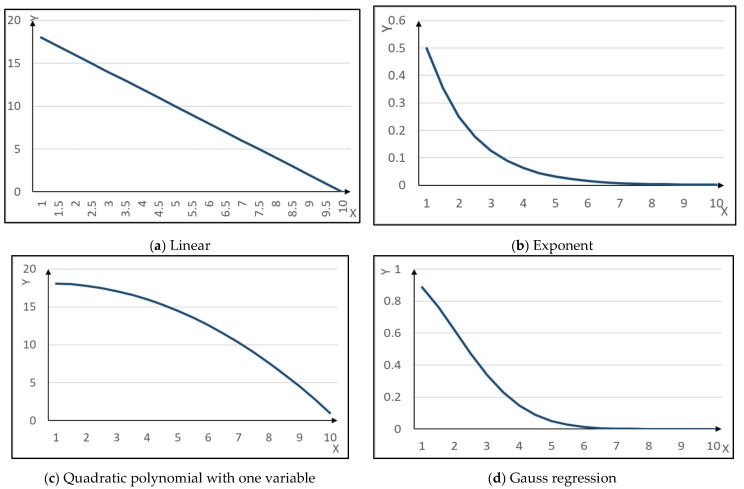
The mathematical model.

The measured voltage output of the sensor is theoretically correlated with the humidity level of the grain medium. Consequently, given the recorded sensor voltage, the humidity level of the grain medium can be ascertained. By embedding the sensor within the grain medium (*Z*) of known humidity (*W*), the voltage (*U_X_*) across the sensor probe can be measured, as illustrated in Figure 4. Utilizing the acquired data, a mathematical model for monitoring voltage variations corresponding to changes in grain moisture content is established. Figure 5 shows four commonly representative mathematical models (linear, exponential, quadratic polynomial, and Gaussian regression). As seen from Figure 4, the voltage sensed by the sensor is monotonically negatively correlated with the humidity of the medium. Furthermore, as the humidity of the medium increases, the rate of voltage change captured by the sensor gradually decreases. Therefore, through comparison, the quadratic polynomial function exhibits the most similar pattern to the sample curve in Figure 4. Consequently, the quadratic polynomial equation is chosen as the fitting model, and the specific formula is provided below:

(7) $$W=a{\left(U_{X}+b\right)}^{2}+c=a{\left[(K_{f}*U_{B}*R_{0})/R_{x}+b\right]}^{2}+c$$

In this context, *a* represents the coefficient, *b* signifies the offset of *x*, and *c* denotes the offset of the vertical coordinate. Consequently, in order to resolve the humidity calibration model, the parameters *a*, *b*, and *c* must be determined initially.

**Figure 6:**
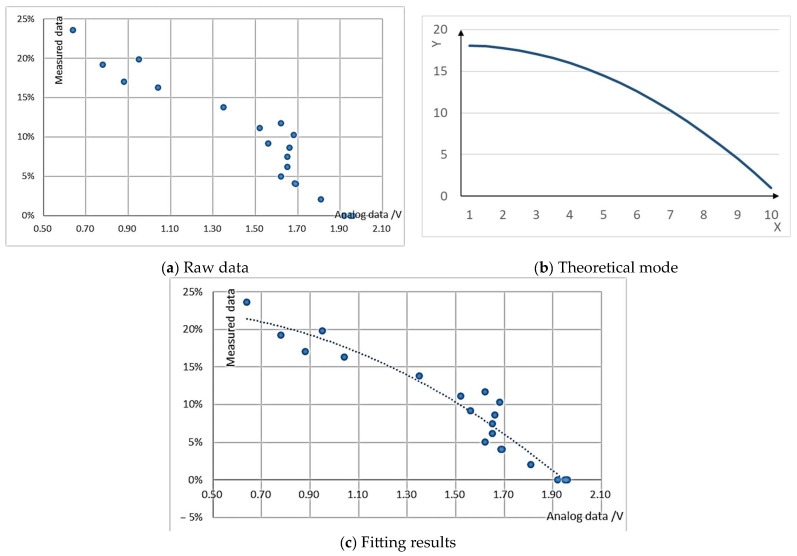
The example of raw data and fitted model.

Using red beans as a case study, the raw data and the corresponding fitted model are illustrated in Figure 6a and Figure 6b respectively. The ultimate outcome of the fitting function is presented in Figure 6c.

Due to variations in grain mediums, the specific parameters of the calibration fitting model for different grain moisture levels also differ. Solely employing the aforementioned method to acquire calibration models results in high costs, extensive workload, and low efficiency. To address this issue, this study proposes the utilization of support vector machine (SVM) models to process experimental data. This approach is employed to obtain humidity calibration models for different grain mediums.

SVM is a supervised learning model commonly utilized for pattern recognition, classification, and regression analysis. It can incorporate kernel functions to enable nonlinear classification and regression. In regression tasks, the model endeavors to fit as many data points as possible within a margin range while constraining margin violations. In essence, it adjusts parameters to maximize the positioning of sample points within the margin range. Addressing the specific characteristics of the data in this study, a polynomial kernel function is introduced to map the original data into a higher-dimensional space. As mentioned earlier, the humidity calibration fitting model is a quadratic equation, thus the kernel function is a quadratic kernel. Given that *K*(*x*,*z*) is a function or positive definite kernel, there exists a mapping Φ from the input space to the feature space such that for any *x*, *z*:

(8) $$K(x,z)=\Phi {\left(x\right)}^{T}•\Phi (z)$$

In the high-dimensional feature space, the inner product of *x* and *z* can be substituted with the kernel function *K*(*x*,*z*). Accordingly, the solution is derived as a nonlinear support vector machine for:

(9) $$f(x)=sign(\sum_{i=1}^{n}\alpha_{i}^{*}y_{i}K(x,x_{i})+b^{*})$$

Here, *f*(*x*) represents the prediction outcome for sample *x*, the *sign* denotes the sign function, *α_i_* is the Lagrange multiplier corresponding to the support vector, *y_i_* is the label associated with the support vector, *K*(*x*,*x_i_*) signifies the kernel function, and *b* represents the model bias.

This method allows for the rapid determination of various parameters of the grain moisture calibration model. It possesses advantages such as speed, efficiency, and precision.

According to Figure 2, a vertical cylindrical pillar is installed at the center of the grain bin. Humidity sensors are uniformly mounted on this pillar. Taking a typical large-scale storage bin as an example, with a height of 20 m, the sensors are installed in five layers. The bottom layer is positioned 20 m away from the grain inlet. The distance between each sensor within a layer is 4 m. Each layer contains eight humidity sensors, positioned at angles of 45° from each other. If the height of the grain bin varies, the spacing between sensors in each layer can be adjusted accordingly, or the number of sensing layers can be reduced as needed. By processing the humidity information collected by the sensors, various data such as the average humidity, maximum humidity, minimum humidity for each layer, or humidity variation at different depths can be obtained, ultimately providing comprehensive humidity information within the storage facility.

(10) $$W_{\max\_i}=\max(W_{i\_1},W_{i\_2},W_{i\_3},\ldots W_{i\_n})$$

(11) $$W_{avg\_i}=\frac{\sum_{j=1}^{n}W_{ij}}{n}$$

(12) $$W_{avg\_j}=\frac{\sum_{i=1}^{m}W_{ij}}{m}$$

(13) $$W_{avg}=\frac{\sum_{i=1}^{m}\sum_{j=1}^{n}W_{ij}}{m*n}$$

Here, *n* represents the vertical layers of sensors, while *m* denotes the number of sensors per layer; *W_max_i_* indicates the maximum humidity within the *i* layer, *W_avg_i_* denotes the average humidity among the n sensors within the *i* layer, *W_avg_j_* represents the average humidity vertically across the *j* sensor within each layer, and *W_avg_* signifies the average humidity across all sensors within the entire storage device.

## 4. Sensor Design and Simulation

To enhance the efficiency of sensor design, theoretical simulation analysis is conducted on the material, length, and spacing of sensor probes. The material and structure of the probes are crucial for the sensing accuracy and effective monitoring range of the sensor.

### 4.1. Materials Selection

Considering the application environment of the sensors, we have placed high demands on the hardness, toughness, and corrosion resistance of the sensor probes. It is known that the main materials for current humidity sensor probes include stainless steel, copper, and polymers. Additionally, researchers often use ceramic materials to manufacture insulation and protective layers for sensors to prolong the probe’s lifespan. Key attribute information of some commonly used materials is provided in Table 2.

Humidity sensors are often subjected to complex environments characterized by high humidity and temperature, placing high demands on both the sensor’s corrosion resistance and material stability. Moreover, in large-scale grain bins, changes in grain quantity exert force on the sensor probes, leading to probe deformation. Therefore, to prolong the sensor’s lifespan, the probe material must possess high hardness and toughness. After comprehensive comparison of various material properties, 316 stainless steel with a resistivity of 0.81 × 10^−6^ was ultimately selected as the probe material for this study.

### 4.2. Simulation of Probe Length

**Figure 7:**
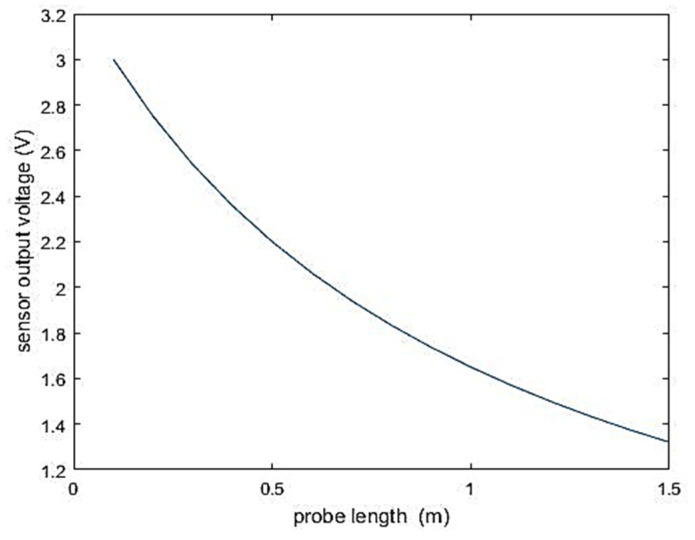
The sensor output voltage vs. probe length (constant measurement medium).

The moisture information sensed by the sensor originates from the medium within the probe. Therefore, the higher the proportion of the medium in the middle of the probe to the total medium, the more representative the data information obtained by the sensor. Extending the effective sensing range of the sensor is crucial for improving the accuracy of equipment information acquisition without compromising the monitoring accuracy of the circuit. In this study, Matlab 2016b was used to simulate the detection effects of sensor probes of different lengths with fixed medium and probe spacing. This analysis aimed to investigate the influence of sensor probe length on the sensing range and accuracy of the sensor. The probes were made of 316 stainless steel, with a diameter of 2 mm and spaced 5 cm apart. The simulation results of the sensor are shown in Figure 7.

In the absence of external force interference, the stable output voltage range of the sensor is between 1.8 V and 2.3 V. As depicted in Figure 7, under these conditions, the optimal length of the sensor probe is determined to be 0.8 m.

**Figure 8:**
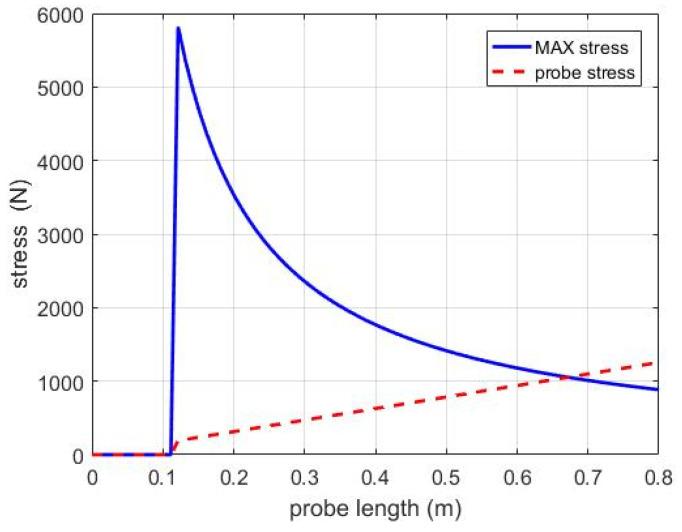
The theoretical analysis of force on the probe.

However, in practical applications, sensors installed in warehouses are subjected to vertical forces from the grains. Therefore, further analysis of the force acting on the sensor probe is necessary. The results are shown in Figure 8. Under ideal conditions, when the force applied to the probe tip reaches 883.88 N, the probe shape undergoes deformation. With wheat density typically ranging from 700 to 800 kg/m^3^, assuming an average density of 800 kg/m^3^, and considering the sensor positioned 20 m below the grain fill level, Newton’s second law is used to calculate the force acting on the probe. At this point, the force acting on the 0.8 m probe tip is approximately 1254.40 N, significantly exceeding the probe’s force limit. Therefore, adjustments to the sensor probe length are necessary based on the force acting on the sensor at the lowest point.

To further clarify the optimal length results, theoretical maximum force and theoretical bottom force for different probe lengths are calculated incrementally at 1 cm intervals. Some of the calculated data are shown in Table 3. From the table, it is evident that the theoretical maximum force for probes below 67 cm exceeds the theoretical bottom force. However, when the length reaches 68 cm, the theoretical maximum force becomes less than the theoretical bottom force. Therefore, the theoretically optimal probe length determined through simulation is 67 cm.

**Figure 9:**
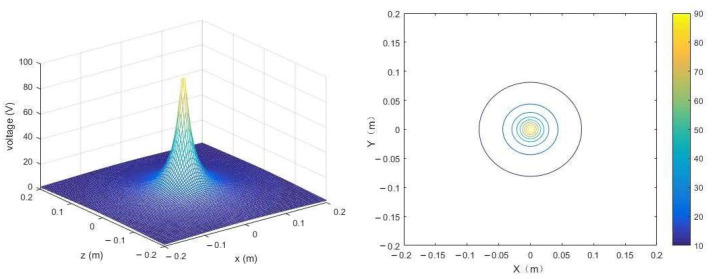
The magnetic field distribution around the probe.

**Figure 10:**
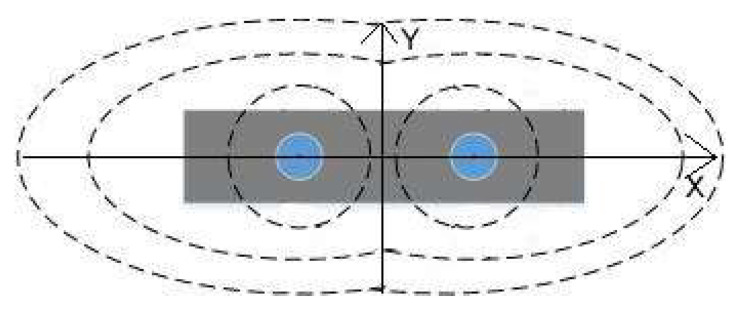
The magnetic field distribution around the sensor (Side view). **Note:** The gray box represents the insulated wrapped portion of the sensor. The blue circle is the sensor probe. The dashed line is the magnetic field around the probe.

To clarify the installation details of the sensor, the voltage distribution around the probe in an ideal environment is constructed using Matlab 2016b, as depicted in Figure 9. The voltage around the probe decreases with increasing distance, as depicted in the Figure 10. As previously mentioned, the voltage influence range in the horizontal direction (*X*) of the probe will be greater than in the vertical direction (*Y*). Therefore, to reduce interference between sensors, it is recommended to install the sensors with the probe plane parallel to the ground.

## 5. Experiments

### 5.1. Experimental Design

**Figure 11:**
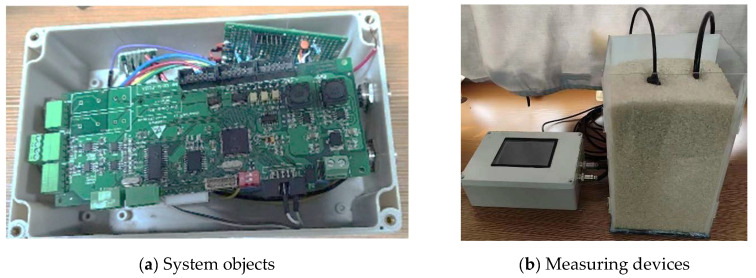
The test system.

This paper presents a design method for a grain humidity sensor based on the standing wave method. To enhance the monitoring performance of this sensor, we constructed a prototype of a grain medium humidity monitoring system, as depicted in Figure 11. Furthermore, to validate the accuracy of the moisture information obtained by the sensor, a drying control experiment was designed as follows.

This study intends to monitor the RH (relative humidity) levels through experimental data pairing. The specific method involves comparing and analyzing the data obtained from the sensor with the real humidity data obtained through the drying and weighing method. Utilizing SVM (support vector machine) model, a moisture calibration model is constructed. As mentioned earlier, the optimal storage humidity for grains generally falls between 10% and 15%. Additionally, during the experimental process, it was observed that when the humidity of grains reaches 20%, it leads to water accumulation, subsequently causing severe mold or decay of the grains. Therefore, a measurement range of 0% to 35% can meet the requirements for daily grain storage. Furthermore, while expanding the detection range of the sensor to ensure accuracy, it necessitates the addition of circuit components, which would increase the research and development costs. This contradicts the aim of this paper to develop a simple, easy, and low-cost humidity sensor. So, the experiment is conducted within the range of grain moisture content from 0% to 35%, and the specific experimental design is as follows:

1. Experimental materials;

To meet the requirements for establishing calibration models for different grain moisture levels, common grains such as millet, rice, and wheat were specifically selected as experimental materials.

2. Experimental apparatus;

The equipment used includes a high-temperature aging chamber, an electronic scale with one-thousandth precision, standardized semi-transparent containers, a vernier caliper (0.02 mm), developed sensors, and the MAS-1 humidity sensor.

3. Experimental procedure;

- Preparation of materials;

The experimental materials selected were millet, rice, and wheat. The original samples were placed in a high-temperature aging chamber and baked for 8 h to remove excess moisture from the samples. Subsequently, 5000.00 g of the dried samples were weighed using an electronic scale and placed into uniformly sized cubic containers for experimentation. Following the aforementioned procedure, three samples each of millet, rice, and wheat were prepared. Controlling the surrounding environment ensured that the samples remained in a stable environment at 25 °C.

- Data collection experiment;

Using an electronic scale, an appropriate amount of distilled water (25 °C) was measured. After recording the data, all the water was added to the dried samples. Following thorough mixing of water with the grains, the sensor was vertically inserted into the central position of the sample under test. We ensured that the sensor probe was fully immersed in the grains without touching the edges of the container and maintained consistent depth and position with each insertion. The output data of the sensor and the moisture data of the grains were recorded. This experimental procedure was repeated to obtain sensor data at different moisture levels.

- Comparative experiment;

Using the same method, data from the MAS-1 sensor were simultaneously collected, and proper data recording was conducted. Subsequently, SVM was employed to construct the humidity calibration model. The accuracy of the humidity calibration models for both sensors was then verified.

- Validation experiment;

The theoretical data calculated by the model were compared and analyzed against the actual moisture data to validate the authenticity and reliability of the model.

### 5.2. Result and Analysis

#### 5.2.1. Experiment Result

**Figure 12:**
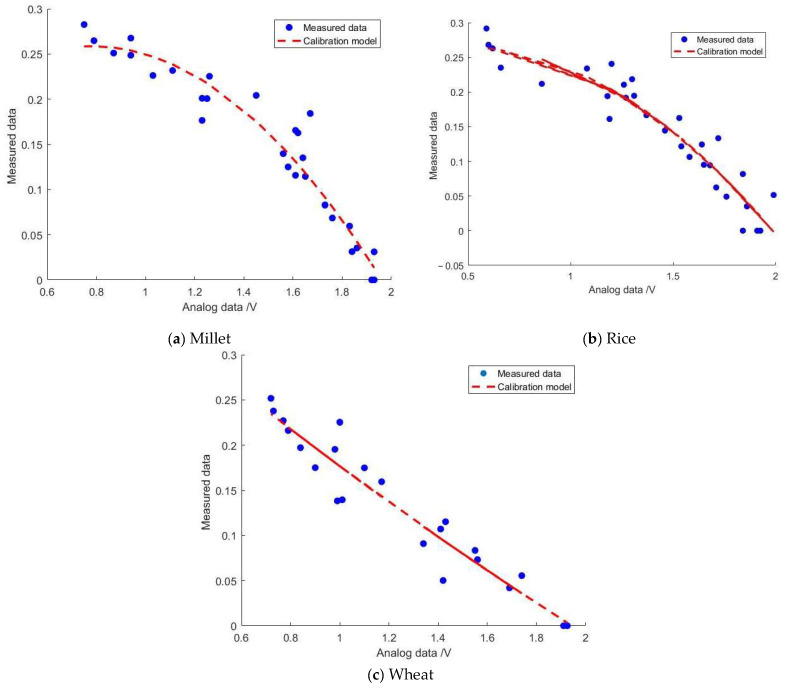
The moisture calibration of different granular media (developed sensor).

**Figure 13:**
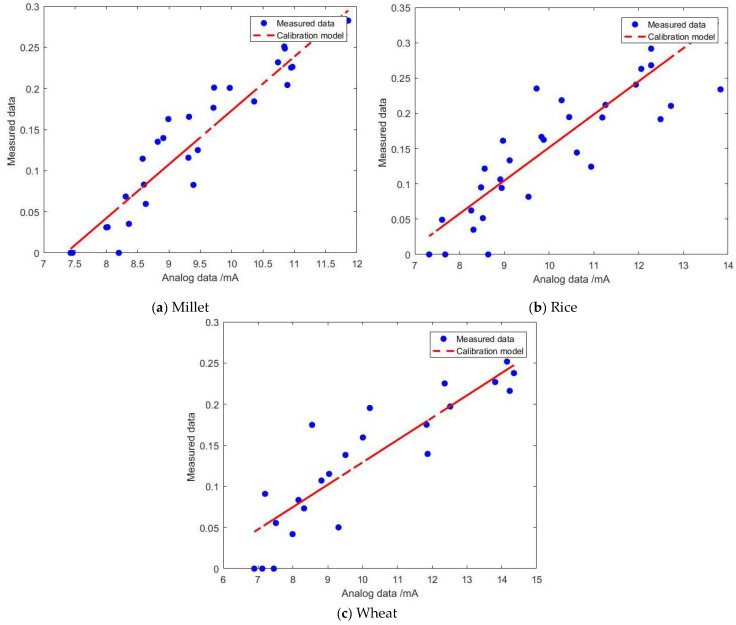
The moisture calibration of different granular media (MAS-1).

The moisture calibration models for the developed sensor and the MAS-1 sensor were constructed using SVM. The results are depicted in Figure 12 and Figure 13. The horizontal axis represents the analog information collected by the sensors, while the vertical axis represents the actual moisture content of the grain medium. The output signal of the developed sensor is voltage (V), whereas the output information of the MAS-1 humidity sensor is current (4–20 mA). As mentioned in Section 3.2, the humidity calibration model of the developed sensor exhibits nonlinearity, thus necessitating the use of a kernel function in the SVM model:

(14) $$K(x,x_{i})={\left(\gamma \times (x\cdots x_{i})+r\right)}^{d}$$

In the equation provided, *γ* represents the scaling factor of the kernel function, *r* stands for the constant bias, and *d* denotes the maximum number of iterations. Here, *x* and *x_i_* represent the feature vectors of the samples. Initially, the kernel matrix *K* is computed based on the features of the training data:

(15) $$K(x_{i},y_{j})={\left(\gamma \times (x_{i}\cdots x_{j})+r\right)}^{d}$$

Here, (*x_i_* · *x_j_*) denotes the inner product of samples *x_i_* and *x_j_*. The next step involves constructing an *m × m* kernel matrix *K* by computing the kernel function values between each pair of samples in the training set, where *m* represents the number of training samples. Following this, the Lagrangian function of the original problem is:

(16) $$L(w,b,\alpha )=1/2||w|{|}^{2}-\sum \left(\alpha_{i}\times (y_{i}\times (w\cdots x_{i}+b)-1)\right)$$

In the provided equation, *w* is the weight vector, representing the model coefficients, *b* stands for the bias, *α* is the Lagrange multiplier, *y_i_* represents the label of the training sample, and *x_i_* corresponds to the features of the training sample.

Subsequently, the Lagrangian dual problem is formulated, and a convex optimization algorithm is employed to determine the Lagrangian multipliers, *α*. Each *αi* must adhere to the following constraints:

(17) $$\left\{\begin{array}{l} \alpha_{i}>0\\ \sum \left(\alpha_{i}*y_{i}\right)=0 \end{array}\right.$$

Upon obtaining the solution to the optimization problem, the corresponding *α* values associated with the support vectors are derived. Subsequently, the model’s bias, denoted as *b*, is computed. To calculate this, a subset of the support vectors is selected. For a specific support vector (*x_i_*, *y_i_*), the bias *b* is computed as follows:

(18) $$b=y_{i}-\sum \left(\alpha_{i}\times y_{i}\times K\left(x_{i},\;x\right)\right)$$

Ultimately, the coefficients (*w*) of the model are computed to represent the normal vector of the decision boundary.

(19) $$w=\sum \left(\alpha_{i}\times y_{i}\times \varphi \left(x_{i}\right)\right)$$

where *α_i_* represents the Lagrange multiplier corresponding to the support vector, *y_i_* denotes the category label associated with the support vector, *φ*(*x_i_*) signifies the mapping function of the sample features, and *x_i_* stands for the eigenvector of the support

Building upon the aforementioned procedures, distinct moisture calibration models were established for millet, rice, and wheat, outlined as follows:

(20) $$\begin{array}{l} y_{1}=-0.1859x^{2}+0.2905x+0.1451\\ y_{2}=-0.1162x^{2}+0.1108x+0.2381\\ y_{3}=0.0159x^{2}-0.234x+0.3948 \end{array}$$

Here, *y*_1_ represents the humidity calibration model for millet, *y*_2_ denotes the humidity calibration model for rice, and *y*_3_ signifies the humidity calibration model for wheat. The determination coefficients (R^2^) for the three calibration models are as follows: R_1_^2^ = 0.9342 for millet, R_2_^2^ = 0.9023 for rice, and R_3_^2^ = 0.92621 for wheat. Notably, all of these values exceed 0.9, indicating strong model performance.

From Figure 13, it can be observed that the analog information obtained by the MAS-1 humidity sensor in the three types of grains, namely millet, rice, and wheat, exhibits a monotonically positive correlation with moisture content. The moisture calibration formula satisfies a linearly increasing relationship. At this point, the coefficient *w* of the model satisfies:

(21) $$w=\sum (\alpha_{i}\times y_{i}\times x_{i})\;$$

Therefore, the moisture calibration model is constructed as follows:

(22) $$\begin{array}{l} y_{1}^{\prime }=0.065x-0.4814\\ y_{2}^{\prime }=0.0469x-0.3174\\ y_{3}^{\prime }=0.0272x-0.1424 \end{array}$$

Here, *y*_1_′ represents the humidity calibration model for millet, *y*_2_′ denotes the humidity calibration model for rice, and *y*_3_′ signifies the humidity calibration model for wheat. The coefficients of determination (R^2^) for the three model sets are as follows: R_1_^2^′ = 0.89897, R_2_^2^′ = 0.70336, and R_3_^2^′ = 0.78696. It is observed that the fit is comparatively weaker than that of the developed sensor.

#### 5.2.2. Verification and Analysis

**Figure 14:**
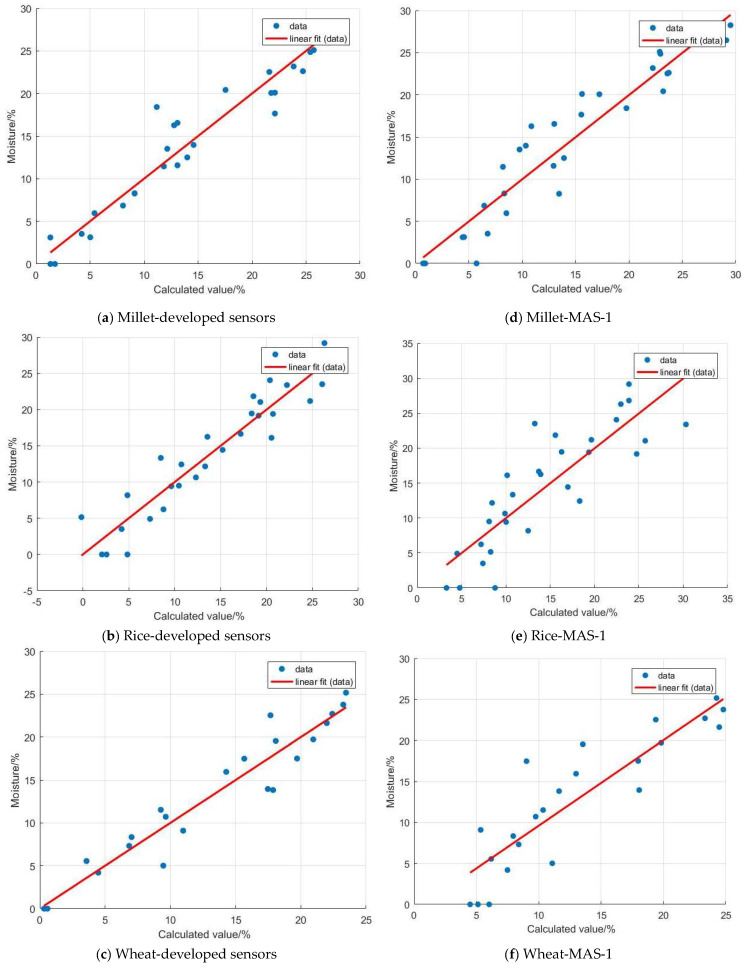
The effectiveness of fitting calculated data to measured data.

The calculated moisture information from both the developed sensor and the MAS-1 sensor was compared with the measured moisture data for validation analysis. The results are shown in Figure 14, where the horizontal axis represents the calculated moisture information from the models, and the vertical axis represents the measured moisture information. From the graph, it is evident that the moisture calibration model’s calculated data for millet, rice, and wheat exhibit a good linear relationship with the measured data. These can be fitted and validated using the equation y = kx + b, where y represents the measured mass moisture content (%), x represents the moisture content of the medium obtained from the model (%), k represents the regression coefficient, and b represents the constant term. The parameters of the validation models for each sensor are presented in Table 4.

From Table 4, it is evident that the coefficient k of the fitting formulas for both sensors is very close to 1, and the constant term b is extremely close to 0. However, the fitting performance of the developed sensor (R^2^ > 0.9) surpasses that of the MSA-1 humidity sensor (0.7 < R^2^ < 0.9). The experimental results indicate that the developed sensor exhibits higher accuracy and is more suitable for measuring grain moisture.

**Figure 15:**
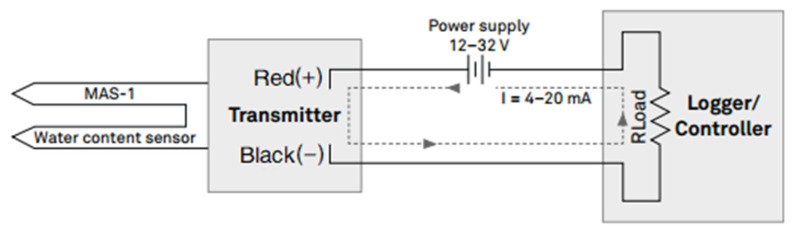
The appearance of the MAS-1 sensor.

The MAS-1 is an integrated sensor and 4 to 20-mA transmitter. The appearance of the MAS-1 sensor is shown in Figure 15. When the MAS-1 is powered by the power supply, it transmits a current though the loop that is proportional to the grain dielectric permittivity and therefore the grain volumetric water content (VWC). First, the grain adjacent to the sensor surface has the strongest influence on the sensor reading and that the sensor measures the VWC. Therefore, any air gaps or excessive grain compaction around the sensor can profoundly influence the readings. As shown in Figure 1, there are many voids in the grain. This is especially true for grains with larger individual volumes, so voids of wheat are larger than those in millet. Consequently, when the MAS-1 sensor measures grain moisture information, the larger the grain particle volume, the greater the measurement error. In contrast, the developed sensor utilizes SVM detection principles to obtain moisture information from the medium, which is less affected by voids. Additionally, the probe length and spacing of the MAS-1 sensor are much smaller than those of the developed sensor (the probe length of the MAS-1 sensor is 5.5 cm, and the probe spacing is 0.5 cm). This means that the effective sensing range perceived by the MAS-1 sensor is much smaller than that of the developed sensor. To summarize, the developed sensor is more suitable for humidity detection in grain media.

## 6. Conclusions

The aim of this study is to develop a grain moisture sensor capable of meeting the humidity monitoring requirements for large-scale, long-term grain storage. We propose a method for developing a grain medium humidity monitoring sensor and construct humidity calibration models for three main types of grains. These methods have been applied and validated in grain media such as millet, rice, and wheat. To investigate this device, we first analyze the physical structure of the probe under ideal conditions, including the probe’s material, stress, and surrounding voltage changes. The results indicate that the suitable material for the sensor probe is 316 stainless steel, and under ideal conditions, the suitable length of the sensor probe with a spacing of 5 cm is 0.67 m. Secondly, SVM is employed to construct humidity calibration models for millet, rice, and wheat. The test results are satisfactory, with R2 values exceeding 0.9 for all three models, surpassing the MAS-1 sensor in accuracy. Finally, we verified the accuracy of the model. The computational results of the three sensor models align well with the validation results from actual data (all R^2^ > 0.9), outperforming the MAS-1 sensor.

The limitation of this study is that the developed sensor system only enables the monitoring of grain humidity information, without involving the integrated control of devices such as ventilation and heating within the storage unit. In future research, we will focus on the control facilities within the storage unit to construct an optimal humidity control model for grain storage. We aim to provide the optimal storage conditions for target grain crops, thereby prolonging storage time while reducing nutrient loss. The grain humidity sensor provided by this study can offer reliable data support for the intelligent management of grain warehouse storage environments.

## Figures and Tables

**Table 1:** Research results of humidity sensors (Undivided).

Devices	Target Media	Sensor Principle	Model Improvement	Advantages	Ref.
New Fringe Field Capacitive (FFC) Sensor	Wheat.	Capacitance method	Evaluating Potential Best-Fit Polynomial Models with Linear Coefficients Using the GLM Procedure of SAS	Suitable for bulk containers; high precision;	[24]
Multi-Grain Moisture Measurement Sensor Based on Capacitive Sensing Technology	Mustard, Chickpea, Urad and Wheat	Capacitance method	Determination of Grain Moisture Using Empirical Calibration Equations	Introduction of thermistor improves accuracy and stability of oscillation unit	[25]
Grain Equilibrium Moisture Content (EMC) Measuring Instruments	Corn	Measurement of equilibrium water content based on air humidity	/	Portable; high accuracy; low cost ($85)	[26]
Capacitive Corn Moisture Sensor	Corn	Capacitance method	Series capacitance calculation method; correlation of capacitance measurement circuit resolution with moisture sensor resolution;	Considers the effect of porosity change on water content measurement; can be applied to large storage	[27]
Microwave Moisture Sensing System for Granular Materials in Arbitrarily Shaped Containers Based on Wet Box Scanning	Red bean, Black bean, Mung bean, rice, Peanut and Soybean	Microwave technology	Optimization of moisture models using microwave attenuation and thickness of the measured medium	Higher accuracy; high efficiency; can be applied to humidity detection of containers of arbitrary shapes	[37]
Microstrip Coupled Line Sensor	Rice	Microstrip transmission line sensors	/	Relatively low cost	[38]
Potato Peel-based Humidity Sensor (PPHS)	Noncontact screen panel, Respiratory rate, Sensing humidity content of real environment, and for Skin care	Electrical resistance	Boltzman curve fitting	High precision; low cost; pollution-free; simple and efficient	[44]
Triboelectric nanogenerator (TENG)	Generating point, Sensing mechanical movement and Humidity	Capacitive or piezoresistive technologies	/	Easy; low-power consumption	[45]
Fully biocompatible gelatin-based moisture sensor	Environmental monitoring, Health monitoring, Food storage, Touchless sensing	Electrical resistance	/	It offers potential for addressing environmental concerns associated with toxic materials and versatile humidity sensing capabilities.	[46]
High-sensitivity moisture sensor based on natural hydroxyapatite	Environmental monitoring, Pharmaceutical industry, Agricultural sector	Capacitance method	/	High sensitivity; fast response with a short recovery time; excellent reproducibility; high selectivity	[47]
High-Performance Triboelectric Nanogenerators	Create energy-harvesting, Health-monitoring devices and Tactile, Humidity-sensing applications	Voltage method	/	Sustainable solution for energy generation and sensing applications	[48]

**Table 2:** Probe material properties (Undivided).

Material	Resistivity (Ω·m)	Conductivity	Hardness	Toughness	Corrosion Resistance	Stable
Copper	1.68 × 10^−8^	Excellent	Low	High	Excellent	Very active
Iron	10–15 × 10^−8^	Good	Higher	Average	Poor	Generally active
Stainless steel 316	0.74–0.81 × 10^−6^	Poor	Higher	High	Excellent	Inactive

**Table 3:** Maximum and bottom forces for different probe lengths.

No.	Probe Lengths (m)	MAX Stress (N)	Stress (N)
1	0.41	1745.29	635.14
2	0.42	1703.28	651.02
3	0.43	1662.28	666.9
4	0.44	1624.27	682.77
5	0.45	1587.26	698.65
6	0.46	1551.26	714.53
7	0.47	1518.25	730.41
8	0.48	1485.25	746.29
9	0.49	1454.24	762.17
10	0.50	1425.24	778.05
11	0.51	1396.23	793.92
12	0.52	1369.23	809.8
13	0.53	1343.22	825.68
14	0.54	1317.22	841.56
15	0.55	1293.22	857.44
16	0.56	1269.21	873.32
17	0.57	1247.21	889.19
18	0.58	1225.20	905.07
19	0.59	1204.20	920.95
20	0.60	1183.20	936.83
21	0.61	1164.19	952.71
22	0.62	1145.19	968.59
23	0.63	1126.19	984.47
24	0.64	1108.18	1000.34
25	0.65	1091.18	1016.22
26	0.66	1074.18	1032.10
27	0.67	1058.18	1047.98
28	0.68	1042.17	1063.86
29	0.69	1027.17	1079.74
30	0.70	1012.17	1095.62

**Table 4:** Fits to calculate the true value of the data are fitted.

Sensors	Grain	k	b	R^2^
Developed sensors	Millet	0.9998	0.00005	0.9342
	Rice	0.9998	0.00004	0.9023
	Wheat	0.9995	0.00007	0.9261
MSA-1	Millet	0.9995	−0.0002	0.8992
	Rice	0.9992	−0.0002	0.7244
	Wheat	1.0436	−0.0082	0.7892

## Data Availability

Data are contained within the article and Supplementary Materials.

## Supplementary Materials

The following supporting information can be downloaded at: https://www.mdpi.com/article/10.3390/s24092854/s1.
