# Supplementary material for: A Design Method for an SVM-Based Humidity Sensor for Grain Storage
Source: Sensors (Basel). 2024 Apr 30;24(9):2854. doi: 10.3390/s24092854 (PMC11086073; doi:10.3390/s24092854)
Supplement: Supplementary file 1 [file sensors-24-02854-s001.zip › sensors-2908814-supplementary.pdf]

Attachment materials of Sensor Design

1. Circuit Design

In this paper, we introduce a grain humidity sensing device utilizing the standing wave ratio method alongside various grain-specific humidity calibration models. To assess the efficacy of both the device and models, we developed a comprehensive sensing and analysis system. This system is capable of receiving real-time data input from the sensing device, conducting analysis and processing, and ultimately delivering moisture information output.

The schematic diagram illustrating the overall circuit structure of the grain humidity sensor is depicted in Figure S1. This circuit comprises the MSP430 core processor and its associated circuitry, power supply circuit, high-frequency signal generation circuit, sensing circuit, and additional components. The MSP430 core processor and its associated circuitry serve as the sensor's central processing unit, orchestrating the functions of each module to ensure stable sensor operation. The power supply circuit is responsible for providing the requisite DC voltage to various components, ensuring their stable operation. Meanwhile, the high-frequency signal generation circuit generates a consistent high-frequency square wave to furnish stable signals for the sensing circuit. The sensing circuit, a pivotal component, acquires moisture-related information from the grain medium by measuring its voltage characteristics.

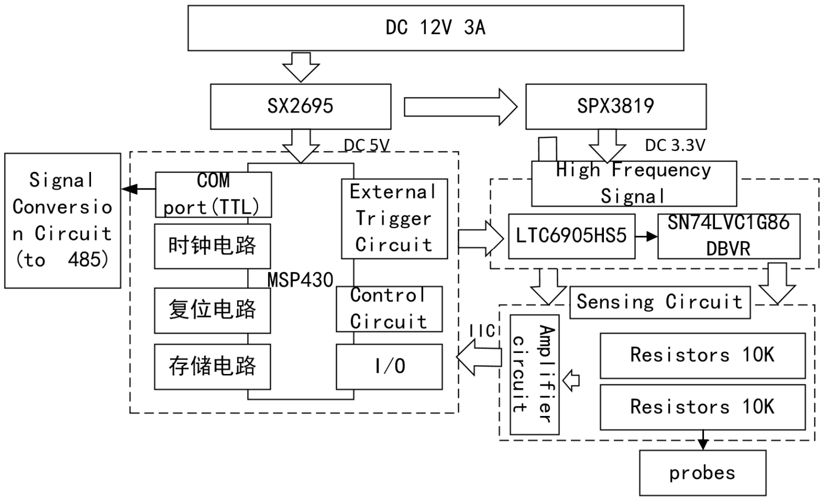

Figure S1. The sensor circuit design diagram.

1. MSP430 core processor and core circuit;

The heart of the sensor lies in its core processor, comprising the MSP430 minimum system alongside reset circuits, clock circuits, storage circuits, and other sub-circuit modules (refer to Figure S2). The sensor system incorporates dual reset circuits to accommodate varying reset demands across different debugging and application scenarios. Furthermore, an independent clock circuit enhances the sensor's time calibration capability by enabling self-powering via a battery, thereby facilitating real-

time synchronous data acquisition. To support the sensor model's self-learning process, historical analog data is stored in the system's Flash memory, providing valuable historical data support.

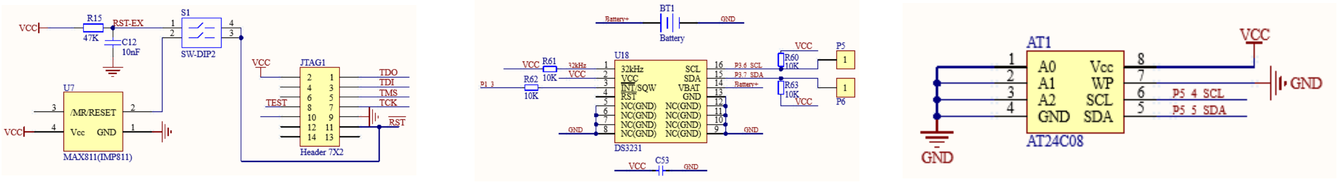

(**a**) Reset circuit (**b**) Clock Circuit (**c**) Memory Circuit

Figure S2. The minimum system(Undivided).

2. Power supply circuit;

The power supply circuit constitutes a fundamental component of the sensor. As illustrated in Figure S3, the system is externally powered by a 12V battery. However, specific components such as the MSP430 core board, external trigger circuit, and storage circuit necessitate a 5V supply, while the high-frequency signal generating circuit and sensing circuit require a 3.3V supply. Consequently, the power supply circuit is divided into two primary sections: a 12V-5V regulated power supply circuit and a 5V-3.3V high-frequency power supply circuit. This design ensures that each module receives a stable, independent voltage supply to facilitate the normal operation of the sensor system.

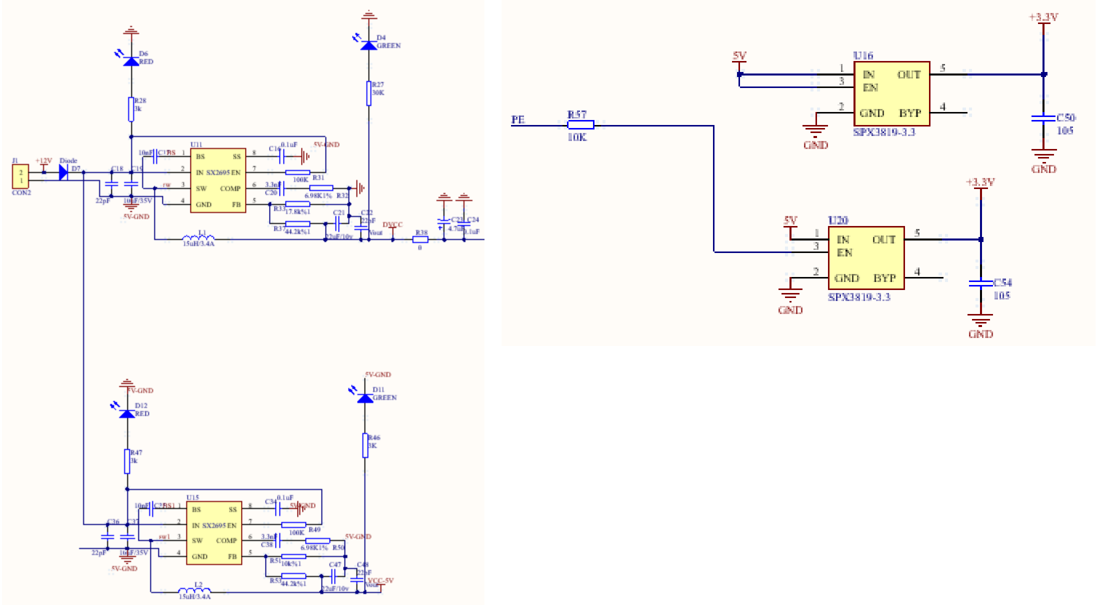

(**a**) 12V to 5V (**b**) 5V to 3.3V

Figure S3. The power supply circuit.

According to Figure S3(a), the power supply circuit employs the SX2695 chip for converting the voltage from 12V to 5V, achieving voltage stabilization through peripheral circuits. The 5V power supply not only energizes the MSP430 chip but also furnishes stable power to auxiliary circuits such as memory, clock, and reset circuits. Despite the MSP430's output pin voltage

being 3.3V, the system utilizes the SX3819 chip, as depicted in Figure S3(b), to convert 5V to 3.3V. This ensures a consistent power supply for high-frequency signal generation and sensing circuits. Furthermore, to mitigate signal interference among modules, the power supply module adopts a segregated power supply approach, guaranteeing independent power provision for each module, thus ensuring their unhindered and efficient operation.

3. Sensing circuit;

The essence of the sensing circuit comprises a high-frequency signal module and a probe, with the former serving as the cornerstone for achieving precise sensing. To mitigate the impact of extraneous factors, such as salt, on the moisture sensing of granular media, a square wave frequency of 100MHz is employed by the system to gauge the moisture content of the media. The system integrates the LTC6905 and SN74LVC1G86 modules to collectively stabilize the high-frequency square wave signal. This configuration guarantees the steadfastness and dependability of the high-frequency signal, thereby fulfilling the criteria for accurately discerning grain humidity.

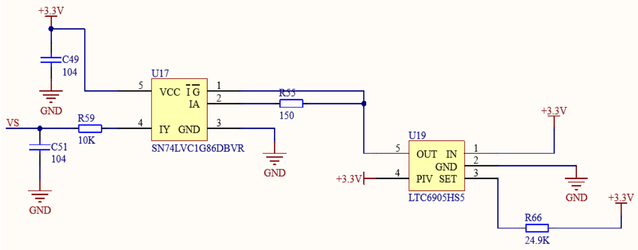

Figure S4. The sensing circuit.

The device employs the LTC6905HS5-100 fixed-frequency silicon oscillator, featuring three vibration frequencies: 100MHz, 50MHz, and 25MHz. Through PIV selection, the module ensures a stable 100MHz frequency square wave output. The load side of the LTC6905HS5-100 connects to the (A) and IA (B) input pins of the SN74LVC1G86 module (refer to Figure S2). The SN74LVC1G86 module functions as a 2-input iso-orthogonal gate arithmetic module, executing the positive logic of the Boolean function $Y = \overline{A}B + A\overline{B}$. To mitigate signal reflection, a 150Ω resistor is placed between IA(B) and the output pin of the LTC6905HS5-100 module. This integration ensures that the SN74LVC1G86 module further stabilizes the square wave signals, preventing any decrease or distortion in square wave amplitude due to overloading, resistor, or capacitor issues, thereby ensuring the device's normal operation.
